# Effects of Charitable Versus Monetary Incentives on the Acceptance of and Adherence to a Pedometer-Based Health Intervention: Study Protocol and Baseline Characteristics of a Cluster-Randomized Controlled Trial

**DOI:** 10.2196/resprot.6089

**Published:** 2016-09-13

**Authors:** Tobias Kowatsch, Jan-Niklas Kramer, Flavius Kehr, Fabian Wahle, Niklas Elser, Elgar Fleisch

**Affiliations:** ^1^ Institute of Technology Management (ITEM) University of St Gallen St. Gallen Switzerland; ^2^ Department of Management, Technology and Economics (MTEC) ETH Zurich Zurich Switzerland; ^3^ CSS Insurance Lucerne Switzerland

**Keywords:** physical activity, self-tracking, adherence, acceptance, pedometer, incentives, digital health intervention, cluster-randomized controlled trial

## Abstract

**Background:**

Research has so far benefited from the use of pedometers in physical activity interventions. However, when public health institutions (eg, insurance companies) implement pedometer-based interventions in practice, people may refrain from participating due to privacy concerns. This might greatly limit the applicability of such interventions. Financial incentives have been successfully used to influence both health behavior and privacy concerns, and may thus have a beneficial effect on the acceptance of pedometer-based interventions.

**Objective:**

This paper presents the design and baseline characteristics of a cluster-randomized controlled trial that seeks to examine the effect of financial incentives on the acceptance of and adherence to a pedometer-based physical activity intervention offered by a health insurance company.

**Methods:**

More than 18,000 customers of a large Swiss health insurance company were allocated to a financial incentive, a charitable incentive, or a control group and invited to participate in a health prevention program. Participants used a pedometer to track their daily physical activity over the course of 6 months. A Web-based questionnaire was administered at the beginning and at the end of the intervention and additional data was provided by the insurance company. The primary outcome of the study will be the participation rate, secondary outcomes will be adherence to the prevention program, physical activity, and health status of the participants among others.

**Results:**

Baseline characteristics indicate that residence of participants, baseline physical activity, and subjective health should be used as covariates in the statistical analysis of the secondary outcomes of the study.

**Conclusions:**

This is the first study in western cultures testing the effectiveness of financial incentives with regard to a pedometer-based health intervention offered by a large health insurer to their customers. Given that the incentives prove to be effective, this study provides the basis for powerful health prevention programs of public health institutions that are easy to implement and can reach large numbers of people in need.

## Introduction

In 2012, noncommunicable diseases (NCD) such as cardiovascular diseases, cancers, respiratory diseases, and diabetes were responsible for 68% of deaths worldwide [1]. Physical activity is known to reduce the risk of various NCDs, including cardiovascular disease, obesity, cancer, diabetes [2,3], as well as of mental illness such as depression [4]. However, it seems increasingly difficult to establish a daily activity routine considering the modern sedentary lifestyle and additional personal (eg, motivation), social (eg, lack of social support), and environmental (eg, time or weather) barriers [5,6]. Indeed, when people’s daily activity is assessed empirically the majority of participants fail to reach activity goals associated with a health promoting lifestyle [7].

The emerging trend of self-tracking [8] and the public interest in self-tracking tools [9-11], offer great potential for providers of disease prevention programs to overcome the barriers to adopting active lifestyles. The health-related benefits of self-tracking tools can be explained by their support of self-regulating processes. For example, a pedometer provides real-time information regarding the number of steps walked per day. By doing so, the pedometer enables its user to monitor and evaluate his or her daily activity, and thus directly supports the user’s self-regulating subfunctions [12]. According to the latter, self-regulation mediates external influences and provides the basis for purposeful action and self-directed change [12]. For example, if one is informed about insufficient physical activity by a pedometer, he or she may decide to go for a walk despite bad weather or to plan the rest of the day in order to reach self-set or given physical activity goals. Consequently, a pedometer may help its user to overcome the abovementioned barriers. A systematic review [13] and a meta-analysis [14] demonstrated the benefits of using pedometers to promote physical activity. Likewise, a metaregression of physical activity interventions found strategies supporting self-regulation to be more effective than other behavioral change strategies [15].

With health care costs being on the rise in Switzerland and other countries [16], health insurance companies are increasingly interested in the potential of pedometer-based physical activity interventions. However, privacy concerns may arise in a health insurance context as pedometers commonly measure very sensitive personal and health-related data besides step counts, such as heart rates, calories, location, and sleep. Privacy concerns seem to almost naturally accompany digitalization in various fields, because the benefits of digitalization often rely on the detection of patterns and correlations in different sources of personal information [17]. Research has addressed privacy concerns in different contexts for example in mobile apps [18,19], location-based services (eg, Google Maps) [20,21], driving behavior [22] or e-commerce transactions [23]. Lack of willingness to disclose personal data has also been identified as one of the main barriers for the digitalization of health care [24]. Privacy concerns have been shown to predict attitudes and behavioral intentions toward health information technology and electronic health care services [25-27]. In a recent study of 333 users of health care wearable devices [27], perceived privacy risks significantly predicted the adoption intention of wearable technology. On the other hand, a large public poll (N=995) illustrates that although 81% of health insurance customers indicated privacy concerns, a substantial proportion (32%) would still be willing to share personal health-related data with their insurance company [11]. These numbers may reflect a phenomenon researchers have titled the privacy paradox [28], namely that people do provide personal data despite expressing concerns regarding their privacy. Research has provided evidence for the privacy paradox for different kinds of information as well as different contexts, such as e-commerce [29] and Web-based shopping [30], finance services [28], and social networks [31]. Norberg and colleagues [28] demonstrated, for example, that in different market-research scenarios involving banks and pharmaceutical companies, participants disclosed significantly more pieces of personal information than they initially intended to disclose. Summarizing the outlined reasoning, it is unclear whether people are willing to participate in a pedometer-based physical activity intervention offered by a health insurance company. A pedometer-based intervention may give rise to privacy concerns, however research indicates that people sometimes do disclose personal information despite being concerned about privacy.

Two different streams of research suggest favorable effects of incentives (eg, financial rewards) when addressing the problem outlined above. First, financial incentives have proven to be beneficial in the context of health behavior interventions. Financial incentive schemes have been effectively used to tackle obesity [32], for smoking cessation [33-35], to increase physical activity [35-37], to promote vaccination [34], and to change many more health-related behaviors [38]. Within physical activity interventions, financial incentives have been shown to increase both performance of participants [36] as well as adherence to exercise sessions [39]. Effects of financial incentives on physical activity have also been assessed for pedometer-based interventions [40-43]. Of those studies, all but one [43] revealed positive effects of financial incentives either on step goal achievement [40,41] or weight loss [42]. However, the effect of financial incentives on performance may only reflect a short-term effect [39] or dissipate as soon as the incentive is withdrawn [44].

Recent research [45] has also considered the effect of charitable incentives as a variation of mere monetary incentives in a pedometer-based physical activity intervention. In contrast to mere monetary incentives, charitable incentives offer the opportunity to donate a specific amount of the received money to a charitable organization. Charitable incentives may thus lead to a sense of moral satisfaction [46] and have so far been typically applied in a marketing context to motivate purchase behavior [47]. However, they have yet to be evaluated in the domain of health behavior and physical activity. In order to contribute to research in this area, we decided to consider financial and charitable incentives in the present study.

Second, rooting in the view of privacy as a commodity [48], most approaches explaining privacy disclosure behavior involve the concept of a privacy calculus (ie, weighting the costs and benefits of sharing personal information [23,24,49]). Specifically, sharing personal health data can be perceived as unfair, if no compensating benefit is provided [49]. Consequently, researchers have tried to augment the benefits of information disclosure by providing financial incentives among others in exchange for personal information [20,48,50-52]. For example, participants in a quasiexperimental setup were more willing to disclose personal information on a website for stock trading when they were offered financial gains [50]. Additionally, two-thirds of participants of a large public survey (N=1100) stated that they expect financial compensation in exchange for providing personal health-related data [53]. Thus, financial incentives can be used to increase the perceived benefits of sharing health-related information.

In conclusion, we assume the benefits of financial incentives to be 2-fold within a physical activity intervention offered by a health insurance company: first, a financial incentive may act as a benefit in the privacy calculus of potential intervention participants, compensating for possible privacy concerns. This effect should be reflected in higher participation rates for experimental groups (EG) in which a financial incentive is provided. Second, in line with previous research, financial incentives may have motivational effects and affect the treatment adherence of participants. Therefore, this study protocol describes the design and methodology in order to examine the effects of the two different incentives on the acceptance of and adherence to a pedometer-based health intervention (PHI). Demographics and baseline characteristics of study participants are presented in the results section. Subsequently, strengths and limitations of the study design are discussed.

## Methods

### Experimental Groups

Over the course of the PHI, participants had to achieve a fixed level of physical activity each month that was tracked using a commercial pedometer device or app that automatically counts the number of steps when walking. In order for the PHI to be effective, 150 minutes of moderate physical activity are recommended [54-56], which on average translates to a goal of 10,000 steps per day [7,57,58]. Upon achieving that goal, participants received a monthly incentive depending on the EG they were assigned to. Textbox 1 shows descriptions of the groups.

**Experimental group descriptions.**

Financial incentive (EG1)

• In this condition, participants were entitled to a $10 reward each month they reached an average of 10,000 steps per day or more. Participants achieving more than 7500 steps per day were granted $5 in order to prevent frustration [7]. The minimum recommendation for daily physical activity is approximately 7500 steps per day [57,58].

Charitable incentive (EG2)

• Here, participants received the same rewards as in the financial incentive condition. However, participants had to decide whether a certain proportion of the money should be donated to a charitable organization chosen from a predefined list (proportions varied from 0% to 100% in steps of 5% with 50% being the default).

Control group (CG/EG1)

• Participants of the control group received no incentives over the first 3 months of the PHI. Due to the practical setting of our study, ethical consent and fair treatment of all participants is of highest relevance. Participants in the control group were therefore entitled to a $20 reward each month they averaged over 10,000 steps per day and a $10 reward each month they averaged over 7500 steps per day over the fourth to sixth month of the intervention. To avoid anticipatory effects on the participation rate, participants in the control group were not informed of the opportunity to receive financial rewards during the second half of the PHI.

Thus, all participants had the chance to earn a maximum of $60 that is paid at the end of the PHI.

### Participant Acquisition and Sample

Customers of a large Swiss health insurance company that met the following requirements were eligible for participation: they had to be at least 18 years old, be registered in a complementary insurance program, accept the participation conditions and privacy terms, and declared to be free of any medical condition that prohibits physical activity. Absence of medical conditions was required in order to avoid potential negative effects on subject’s health due to increased daily activity. In case of uncertainty regarding the health-related eligibility for participation the consultation of a physician was required. Privacy terms essentially stated that only the number of steps will be forwarded to the insurance company for bonus calculation and that data will be analyzed by researchers of the University St. Gallen and ETH Zurich for scientific purposes.

To avoid spill-over effects between the different incentive strategies [45], potential participants were assigned to the different groups based on their canton of residence and invitations were send out after the assignment was complete. As at the beginning of the program, the control group (CG) appeared to be the least attractive condition we wanted to contact a different number of potential participants for the 3 groups according to a proportion of 2 (EG1):2 (EG2):1 (CG/EG1). In order to do so and to further account for differences in activity preferences between urban and rural areas in Switzerland [59], cantons were grouped in blocks of 5 according to number of customers and population density. Each block contained 2 pairs of cantons that were matched for geographical proximity. The matched pairs were then randomly assigned to one of the EGs and the remaining canton was assigned to the control group. To facilitate clustering, cantons with very few customers were combined and treated as one unit. We used the approach of Gao and colleagues [60] for nonaggregate cluster-randomized controlled trials with binary outcomes to estimate the number of participants to be contacted in order to detect an expected difference in participation rate. Being conservative in comparison to public polls [53] and studies [11], we expected a difference of 5% in participation rates (8% participation was expected for both intervention groups vs 3% for the control group). As participants in our study were clustered, a potential design effect had to be considered [60]. An assumed intracluster-correlation of .01 (according to [61]) and the average cluster size of mean (M) = 925 (standard deviation [SD] = 1356) yielded a design effect of 10.24. Thus, in order for the difference in participation rates to be significant at least 15,725 customers needed to be contacted (6475 for each experimental group and 2775 for the control group). We met this requirement by directly contacting 18,638 customers via email before the beginning of the PHI.

### Procedure

After providing consent, all participants were instructed on how to use the pedometer or the app, respectively, and how to share the number of tracked steps via the Web-based platform of the health insurance company. The Web-based platform supported devices of the brands Garmin, Jawbone, and Fitbit, all commonly known manufacturers of wearables and fitness technology. Alternatively, participants could use the Fitbit app that is available for selected mobile phones. A systematic review has confirmed the validity of commercial pedometers [62], and recent studies provide evidence for the accuracy of smartphone apps for tracking physical activity data [63]. Owning an eligible tracking device (pedometer or smartphone app) was thus required for participation. Participants not owning an eligible pedometer were entitled to a 20% discount on a compatible device. All participants could use the Web-based platform any time to gain insight into their physical activity data as well as their degree of achievement with regard to their goal of 10,000 steps per day. In the charitable condition, participants could also log in to choose a particular charitable organization from a predefined list and set the proportion of money they want to donate. Participants were asked to set this proportion once at the beginning and once before the end of the PHI.

During the course of the intervention, participants received short informational texts in order to maintain motivation for daily physical activity (eg, “If you are going by bus consider getting off two stops prior to your destination to reach your goal of 10,000 steps per day”). Those texts were based on information material and recommendations for health effective daily activity provided by the Federal Bureau of Sports as well as on recommendations for increasing step count in everyday life [7] and on the Compendium of Physical Activities 3 [64]. Additionally, participants received a status mail on a monthly basis that informs them once again about their target achievement and the respective amount of money saved or donated during the past month. This mail also contained further season-based tips on how to increase the daily step count (eg, recommending free geocaching apps or websites for summer days or popular snowshoeing trails during winter). The content of the monthly status mails was developed in cooperation with the insurance company.

At any time, participants were able to opt out of the PHI and request the deletion of all submitted data without giving reasons. In order to prevent high dropout rates that have been observed in past pedometer-based interventions [13], no bonus will be granted for past achievements if the participant decides to opt out of the PHI.

### Data Collection and Variables

**Figure 1:**
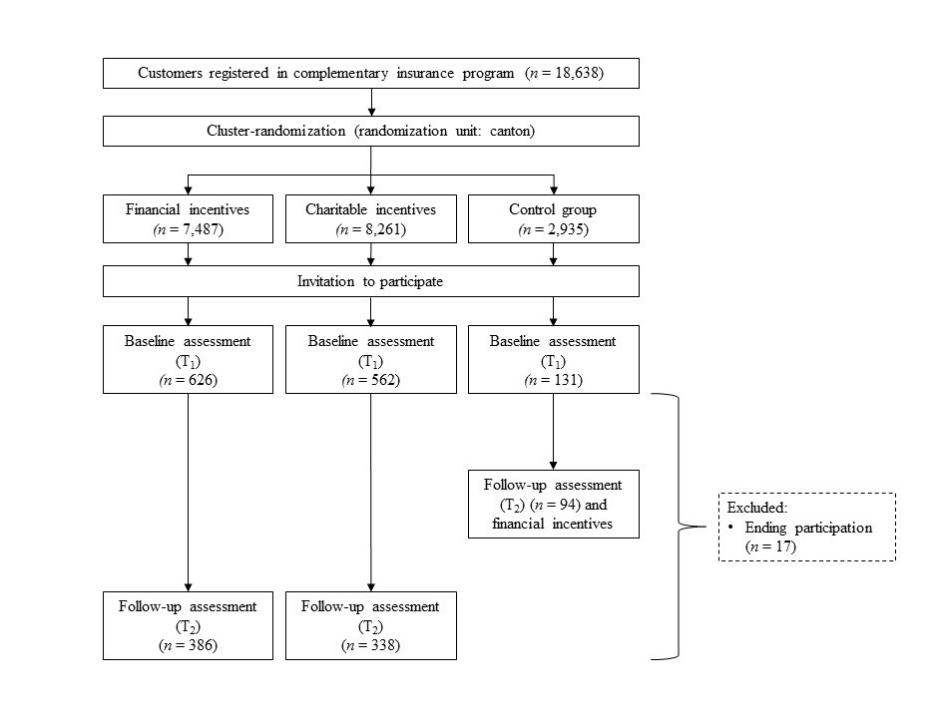
Study design.

Data for analysis is partly collected by submission of information by the participants via the Web-based platform of the insurance company and partly by administering a Web-based questionnaire at 2 different points in time (T_1_ and T_2_) over the course of the intervention. After participants registered their pedometer or smartphone at the Web-based platform of the insurance company, the number of steps were synchronized automatically with the Web-based platform each day at midnight. However, participants could choose to deactivate automatic synchronization and enter their step count manually on the Web-based platform. Days where no step data is available (eg, because the pedometer was not worn or not charged) will be treated as missing data. The first measurement (T_1_) is set at the beginning of the PHI for all groups, whereas the second measurement (T_2_) is set at the end of the intervention for the experimental groups and after the first half of the intervention for the control group before they received financial incentives. Additional data, such as age, gender, or participants’ health service billings, were provided by the insurance company. To guarantee appropriate response rates, participants received additional $5 for each time they completed the questionnaire resulting in an additional bonus of $10. See Figure 1 for an overview of the study design.

The following variables were measured for analysis: the participation rate represents the primary outcome and is measured by calculating the participation rate in total and for the different groups, respectively. Participation rate is defined as the proportion of active participants that is participants that shared their data with the Web-based platform of the insurance company at least once. Secondary outcomes are continued use of the pedometer, performance of the participants, and health condition. The number of days at which participants share their step count with their health insurance company is used as an indicator of the continued use of the pedometer. The number of steps and the amount of money saved or donated indicate the performance of the participants. Apart from the number of steps, physical activity was also assessed by questionnaire measures namely hours of moderate to vigorous physical activity and hours of walking per week at T_1_ and T_2_ (based on the International Physical Activity Questionnaire [65,66]), walking on the way to work, physical activity at work, and during spare time at T_1_ and T_2_. The proportion of money saved versus donated (at T_1_ and T_2_) is further used to evaluate the charitable incentive condition. We use health perception at T_1_ and T_2_ (“How would you rate your overall health status?”) [67-69] and improvement of health perception due to the intervention at T_2_ (eg, “In general, my health improved due to the prevention program”) [67-69] as subjective measures and service billing with the health insurance company (ie, amount of money repaid by the insurance company per participant) after completion of the prevention program and 1 year later as objective measures in order to assess effects on participants’ health condition. Unless otherwise indicated, a 7-point Likert scale, from strongly disagree (1) to strongly agree (7), will be used for items requiring a response scale.

To exclude possible confounding influences, we will measure the following control variables: sociodemographic variables (age, gender, education, income, and nationality [67,70,71]) measured at T_1_, technology readiness [72] measured at T_1_, possession of pedometers and other self-tracking tools at T_1_, pedometer brand measured at T_1_, number of persons living in the participants’ household at T_2_, living environment at T_1_ (city center, outer city, village, countryside), amount of billing services preceding the prevention program at T_1_, exchange with other participants of the prevention program at T_2_, participation of a family member or friend at T_1_, and observation of media coverage of the prevention program and possible impact on participants’ physical activity at T_2_.

Additional variables were measured to better understand the participants behaviour. These variables are participants’ perception of the Web-based platform, perception of the insurance company (eg, perceived social responsibility), customer loyalty, participants’ willingness to share data with their insurance company, willingness to donate (in the charitable incentive group), reasons for participating and not participating, reasons for opting out, and improvement suggestions to the program.

### Data Analysis

Due to the nested structure of the data, mixed-effect models will be used for data analysis. As measurements are nested within participants, the step count measurements represent the level 1 unit of analysis, whereas the participants represent the level 2 unit of analysis. A recent article [73] discusses the problem of faking with regard to financial incentives, (eg, faking step counts in order to qualify for financial rewards). This problem applies to our study as step counts could be entered manually. Step count measurements that are unusually high and were manually entered are likely to represent a tendency of faking. Thus, univariate and multivariate outlier analysis [74] will be conducted in order to identify participants that are prone to faking. Changes on outcomes solely measured at T_1_ and T_2_ will be analyzed performing a repeated-measures analysis of variance (rmANOVA). The analyses will be conducted using the nlme-package [75] in R [76] and the typical significance level of α = 5% will be applied.

## Results

### Survey Participation

In total, 1319 persons participated in the survey at T_1_. Of those, 47.46% (626/1319) belonged to the financial incentives group, 42.61% (562/1319) to the charitable incentives group, and 9.93% (131/1319) to the control group.

### Baseline Characteristics

Table 1 presents the baseline characteristics of all T_1_ survey participants for each of the different EGs. Unfortunately, participants’ service billings with the health insurance company were not yet available at the time of writing. Between-group comparisons are based on one-way ANOVA for continuous variables and on chi-square tests for categorical variables. Due to the large number of participants, even small between-group differences are likely to become statistically significant. Hence, effect sizes are reported for between-group comparisons.

Participants were mostly Swiss (1195/1319, 90.60%), living in a village or on the countryside (836/1319, 63.38%), holding a university degree (597/1319, 45.26%), and were 43-years old on average (M=42.95, SD = 13.11). Slightly more men than women participated in the T_1_ survey (638/1319, 48.14% vs 585/1319, 44.35%). A Fitbit pedometer or the Fitbit app was most often used for tracking physical activity (1116/1319, 84.61%) and more than half of the participants (709/1319, 53.75%) bought a pedometer in order to participate in the PHI.

While baseline characteristics show no meaningful group differences regarding age, gender, education, income, nationality, self-reported physical activity at work and during spare time, walking on the way to work, pedometer brand, prior possession of a pedometer, and participation of a family member or friend, group differences could be observed regarding residence of participants, self-reported physical activity and walking, and subjective health status. Differences regarding residence of participants indicate that matching groups according to population density may not be sufficient to account for residence differences.

Because these baseline characteristics are related to physical activity they are primarily relevant for the analysis of the secondary outcomes of the study. Consequently, residence of participants and subjective health status will be used as covariates in the statistical analyses of the secondary outcomes. Because mixed-effects models will be used for data analysis, group differences regarding baseline physical activity will be directly modelled by allowing different intercepts for the experimental groups.

**Table 1:** Demographics and baseline characteristics.

Charachteristic^a^			Total (*N*=1319)	Financial incentives/ EG1 (*n*=626)	Charitable incentives/ EG2 (*n*=562)	Control group/ CG (*n*=131)	*P*	Effect size^b^
**Group characteristics**								
	Number of cantons		26	8	11	7		
	Number of customers contacted		18,638	7487	8216	2935		
	Population density^c^ (residents/km^2^, median)		233.56	255.15	173.45	221.08		
**Demographic variables**								
	Age		42.95 (13.11)	43.06 (13.25)	42.50 (12.88)	44.37 (13.40)	.36	.002
	Gender (%)						.89	.01
		Female	585 (44.35)	285 (45.53)	244 (43.42)	56 (42.75)		
		Male	635 (48.14)	301 (48.08)	270 (48.04)	64 (48.85)		
		Not declared	99 (7.51)	40 (6.39)	48 (8.54)	11 (8.40)		
	Education^d^ (%)						.17	.10
		University	597 (45.26)	301 (48.08)	244 (43.42)	51 (39.69)		
		Professional School	421 (31.92)	194 (30.99)	188 (33.45)	39 (29.77)		
		High School	219 (16.60)	95 (15.18)	95 (16.90)	29 (22.14)		
		Secondary School	25 (1.90)	13 (2.08)	10 (1.78)	2 (1.53)		
		Primary School	6 (0.45)	4 (0.64)	1 (0.18)	1 (0.76)		
		Not declared	51 (3.87)	19 (3.04)	24 (4.27)	8 (6.11)		
	Place of Residence (%)						< .001	.27
		Town	156 (11.83)	92 (14.70)	49 (8.72)	15 (11.45)		
		Outskirts of town	327 (24.79)	185 (29.55)	116 (20.64)	26 (19.85)		
		Village	644 (48.82)	270 (43.13)	303 (53.91)	71 (54.20)		
		Countryside	192 (14.56)	79 (12.62)	94 (16.73)	19 (14.50)		
	Income in CHF (%)						.25	.11
		< 2500	68 (5.16)	29 (4.63)	35 (6.23)	4 (3.05)		
		2501–5000	203 (15.39)	90 (14.38)	91 (16.19)	22 (16.79)		
		5001–7500	418 (31.69)	204 (32.59)	176 (31.32)	38 (29.01)		
		7501–10,000	220 (16.68)	107 (17.09)	87 (15.48)	26 (19.85)		
		>10,000	137 (10.39)	78 (12.46)	50 (8.90)	9 (6.87)		
		Not declared	273 (20.70)	118 (18.85)	123 (21.89)	32 (24.43)		
	Nationality (%)						.03	.13
		Swiss	1195 (90.60)	554 (88.50)	520 (92.53)	121 (92.37)		
		German	56 (4.25)	36 (5.75)	17 (3.02)	3 (2.29)		
		Other	54 (4.09)	32 (5.11)	16 (2.85)	6 (4.58)		
		Not declared	14 (1.06)	4 (0.64)	9 (1.60)	1 (0.76)		
**Physical activity measures**								
	Self-reported moderate to vigorous physical activity^e^ (hours/week)						< .001	.03
		Mean (SD)	8.90 (11.10)	8.96 (11.38)	8.75 (10.59)	9.26 (11.25)		
		Median	6.00	6.00	6.00	5.25		
	Self-reported walking^e^ (hours/week)						<.001	.03
		Mean (SD)	10.01 (13.70)	10.31 (13.44)	9.99 (15.55)	8.61 (10.87)		
		Median	6.00	6.54	6.00	4.50		
	Physical activity at work		3.45 (1.88)	3.37 (1.84)	3.48 (1.91)	3.67 (1.90)	< .001	.009
	Physical activity during spare time		5.26 (1.17)	5.36 (1.19)	5.19 (1.13)	5.09 (1.22)	.06	.003
	Walking on way to work (%)							
		Yes	234 (17.74)	126 (20.13)	87 (15.48)	21 (16.03)	.10	.06
		No	1085 (82.26)	500 (79.87)	475 (84.52)	110 (84.97)		
**Other**								
	Subjective health status		3.60 (0.73)	3.66 (0.73)	3.55 (0.71)	3.53 (0.80)	<.001	.02
	Pedometer brand (%)						.73	.09
		Fitbit	832 (62.08)	387 (61.82)	359 (63.88)	86 (65.65)		
		Fitbit App	284 (21.53)	141 (22.52)	121 (21.53)	22 (16.79)		
		Garmin	138 (10.46)	69 (11.02)	55 (9.79)	14 (10.69)		
		Jawbone	65 (4.93)	29 (4.63)	27 (4.80)	9 (6.87)		
	Pedometer bought for participation (%)						.04	.07
		Yes	709 (53.75)	316 (50.48)	325 (57.83)	68 (51.91)		
		No	571 (43.29)	289 (46.17)	221 (39.32)	61 (46.56)		
		Not declared	39 (2.96)	21 (3.35)	16 (2.85)	2 (1.53)		
	Participation of family member or friend						.65	.03
		Yes	251 (19.03)	122 (19.49)	108 (19.22)	21 (16.03)		
		No	1068 (80.97)	504 (80.51)	454 (80.78)	110 (83.97)		

^a^ Unless otherwise indicated, mean (SD) are displayed for continuous variables and absolute frequencies (relative frequencies) are displayed for categorical variables.

^b^ η^2^ is used as a measurement of effect size for one-way ANOVAs and Cramer’s *V* is used as a measurement of effect size for chi-square test. Effect size conventions for η^2^ are: .01 (small effect), .09 (medium effect), .25 (large effect). Effect size conventions for Cramver’s *V* are: .10 (small effect), .30 (medium effect), .50 (large effect) for *df*=1 and .07 (small effect), .21 (medium effect), .35 (large effect) for *df*=2 [77].

^c^ Based on information of the Swiss Federal Office for Statistics for the year 2013 [78].

^d^Categories with expected frequencies <5 were not considered for between-group comparison.

^e^ Due to violation of normality a logarithmic transformation was applied for between-group comparison and the median is reported in addition to the mean.

## Discussion

### Strengths and Limitations

This study protocol describes the design and baseline characteristics of a longitudinal cluster-randomized controlled trial testing the effects of monetary and charitable incentives on the acceptance of and adherence to a pedometer-based health prevention program. To the best of our knowledge, this is the first study to systematically test the effects of different incentive strategies within a pedometer-based health intervention offered by a large health insurance company in western cultures. External validity has to be pointed out as a key strength of the described trial. Both study design and incentive strategies are tested in a real-world setting, thus ensuring the applicability of the results and conclusions.

When interpreting the results of this study, some limitations have to be considered: selection effects might affect the participation in the PHI. For example, by especially attracting highly motivated or physically active participants, those effects could potentially undermine the power of our analyses. However, we will be able to control our analyses for prior level of physical activity. Further, comparisons of T_2_ measures between the groups have to be interpreted with caution, because T_2_ reflects different time points for experimental and control groups. T_2_ was set at 6 months after start of the intervention for the EGs and at 3 months for the CG. However, the main focus of this study is on the acceptance of the promotion program, which is operationalized using the participation rate, and is thus not dependent on any T_2_ measurement. Lastly, the goal of reaching 10,000 steps per day on average might have detrimental motivational effects for some participants. It might be perceived as too challenging for very inactive participants or when participants were not able to achieve sufficiently high step counts for several days in a month.

### Conclusions

Considering the importance of physical activity for the course of various NCDs, this study yields important insights for insurance companies, public health institutions, and health practitioners alike. If the effectiveness of the examined incentive strategies is demonstrated, this study provides the basis for simple yet powerful health interventions that can easily be implemented by various health care institutions.

## Abbreviations

CG: control group
EG: experimental group
M: mean
NCD: noncommunicable disease
PHI: pedometer-based health intervention
rmANOVA: repeated-measures analysis of variance
SD: standard deviation
